# Identification of DNA methylation-regulated differentially expressed genes in RA by integrated analysis of DNA methylation and RNA-Seq data

**DOI:** 10.1186/s12967-022-03664-5

**Published:** 2022-10-22

**Authors:** Runrun Zhang, Cen Chang, Yehua Jin, LingXia Xu, Ping Jiang, Kai Wei, Linshuai Xu, Shicheng Guo, Songtao Sun, Dongyi He

**Affiliations:** 1 Department of Rheumatology, Shanghai Guanghua Hospital, Shanghai University of Traditional Chinese Medicine, Shanghai, China; 2 Department of Rheumatology, The Second Affiliated Hospital of Shandong, University of Traditional Chinese Medicine, Jinan, China; 3 Shanghai University of Traditional Chinese Medicine, Shanghai, China; 4 Arthritis Institute of Integrated Traditional and Western Medicine, Shanghai Chinese Medicine Research Institute, Shanghai, China; 5 Department of Medical Genetics, School of Medicine and Public Health, University of Wisconsin-Madison, Madison, WI USA; 6 Department of Orthopaedics, Shanghai Guanghua Hospital, Shanghai University of Traditional Chinese Medicine, Shanghai, China

**Keywords:** Rheumatoid arthritis, Synovial tissue, DNA methylation, RNA-seq, Differentially expressed genes

## Abstract

**Objective:**

To identify novel DNA methylation-regulated differentially expressed genes (MeDEGs) in RA by integrated analysis of DNA methylation and RNA-Seq data.

**Methods:**

The transcription and DNA methylation profiles of 9 RA and 15 OA synovial tissue were generated by RNA-Seq and Illumina 850K DNA methylation BeadChip. Gene set enrichment analysis (GSEA) and Weighted gene co-expression network analysis (WGCNA) were used to analyze methylation-regulated expressed genes by R software. The differentially expressed genes (DEGs), differentially methylated probes (DMPs), differentially methylated genes (DMGs) were analyzed by DESeq and ChAMP R package. The functional correlation of MeDEGs was analyzed by Gene Ontology (GO) and Kyoto Encyclopedia of Genes and Genomes (KEGG). The protein–protein interaction (PPI) network of MeDEGs was constructed by STRING and Reactome FI Cytoscape Plugin. Correlation analysis between methylation level and mRNA expression was conducted with R software.

**Results:**

A total of 17,736 genes, 25,578 methylated genes and 755,852 methylation probes were detected. A total of 16,421 methylation-regulated expressed genes were obtained. The GSEA showed that these genes are associated with activation of immune response, adaptive immune response, Inflammatory response in C5 (ontology gene sets). For KEGG analysis, these genes are associated with rheumatoid arthritis, NF-kappa B signaling pathway, T cell receptor signaling pathway. The WGCNA showed that the turquoise module exhibited the strongest correlation with RA (R = 0.78, *P* = 1.27 × 10^− 05^), 660 genes were screened in the turquoise module. A total of 707 MeDEGs were obtained. GO analysis showed that MeDEGs were enriched in signal transduction, cell adhesion for BP, enriched in plasma membrane, integral component of membrane for CC, and enriched in identical protein binding, calcium ion binding for MF. The KEGG pathway analysis showed that the MeDEGs were enriched in calcium signaling pathway, T cell receptor signaling pathway, NF-kappa B signaling pathway, Rheumatoid arthritis. The PPI network containing 706 nodes and 882 edges, and the enrichment p value < 1.0 × 10^− 16^. With Cytoscape, based on the range of more than 10 genes, a total of 8 modules were screened out. Spearman correlation analysis showed RGS1(cg10718027), RGS1(cg02586212), RGS1(cg10861751) were significantly correlated with RA.

**Conclusions:**

RGS1 can be used as novel methylated biomarkers for RA.

**Supplementary Information:**

The online version contains supplementary material available at 10.1186/s12967-022-03664-5.

## Introduction

Rheumatoid arthritis (RA) is an autoimmune disease characterized by synovitis and system inflammation that can affect many tissues, including the synovial tissue, leading to joint swelling and pain [1, 2]. Its pathogenesis is still not very clear, genetic and environmental have been proved to be the important factors [3]. Epigenetic factors provide a mechanism that links genetics, regulation of gene expression, and environmental risk factors [4]. Epigenetics including histone acetylation and methylation have been identified to be associated with the development of RA [5].

DNA methylation is accomplished by transferring the methyl group from the methyl donor S-adenosylmethionine to the DNA by DNA methyltransferase, leading to the formation of 5-methylcytosine (5-mC). Studies on methylation have focused on the role of 5mC in CpG-rich regions (called CpG islands), where they are enriched in promoters near transcription start sites and their methylation blocks transcription initiation [6].

Transcriptomics is tissue-specific and as such offers an avenue for the investigation of effects localized to cells that are likely to play an important role in the etiology of diseases [7]. RNA sequencing (RNA-Seq) technology has become a primary tool of transcriptomics research to characterize expression within certain cell types and tissues. Illumina methylation BeadChip is a popular method for assaying methylation of CpG sites across the human genome. The Illumina MethylationEPIC (EPIC) array uses the Infinium probe technology and shares 452,453 common probes [8].

**Fig. 1:**
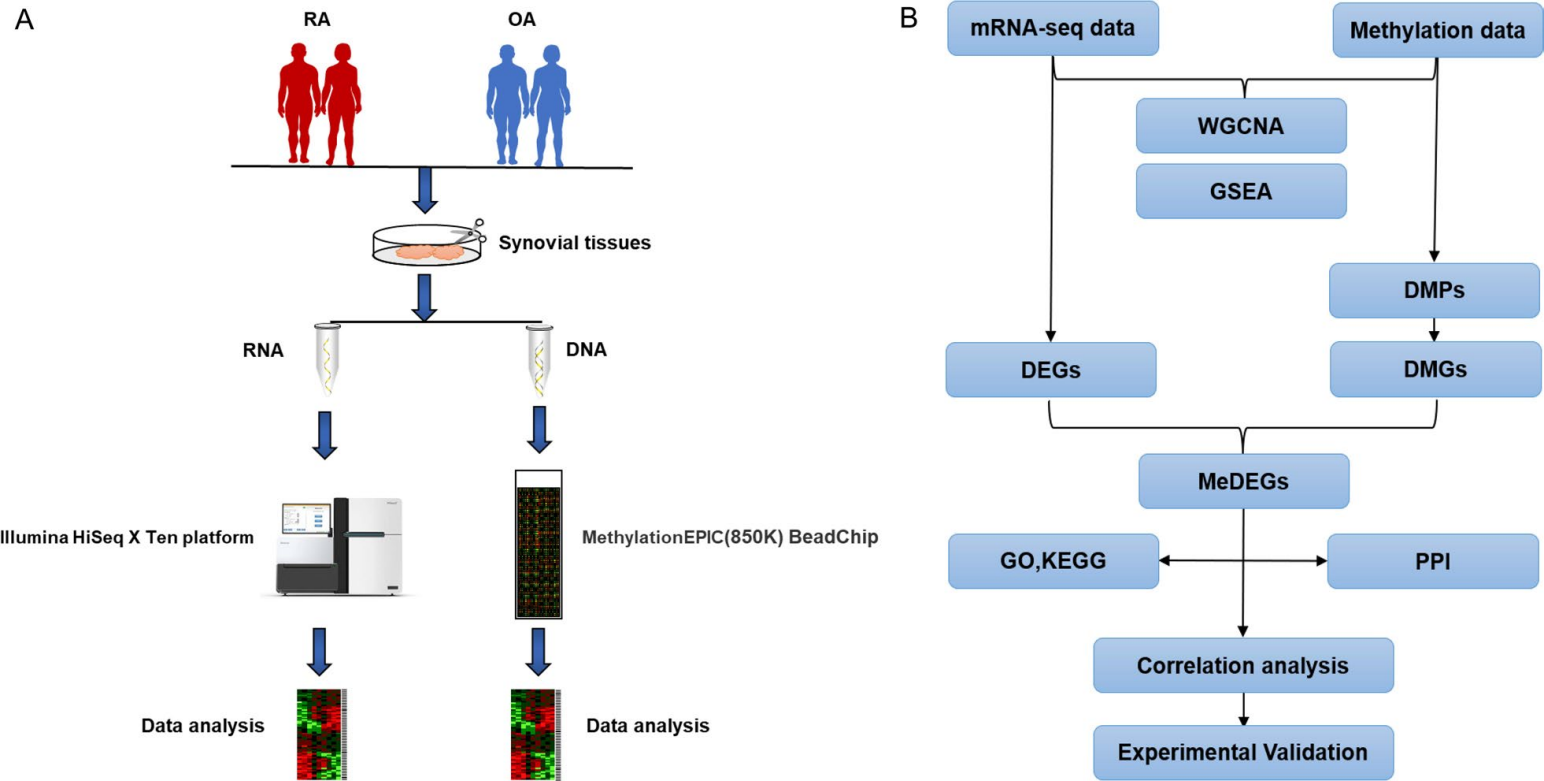
Flowchart for analysis. **A** Experimental flowchart; **B** Data analysis flowchart. RNA-seq: RNA-sequencing; mRNA: messenger RNA; GO: Gene Ontology; KEGG: Kyoto Encyclopedia of Genes and Genomes; DEGs: Differentially expressed genes; DMPs: differentially methylated probes; DMGs: differentially methylated genes; MeDEGs: methylation-regulated differentially expressed genes. WGCNA: weighted gene co-expression network analysis

In the present study, we performed RNA-Seq and 850k methylation array on 9 RA and 15 OA synovial tissues to identify methylation-regulated differentially expressed genes (MeDEGs). The analysis process was shown in Fig. 1. This will be of profound significance for clarifying the role of methylation in RA and identifying the candidate biomarker for RA future research.

## Materials and methods

### Sample collection

Synovial tissue is derived from RA and OA patients after knee joint replacement at Shanghai Guanghua Hospital of Integrated Traditional Chinese and Western Medicine. This study was approved by the Ethics Committee of Guanghua Hospital of Integrated Traditional Chinese and Western Medicine (approval number: 2018-K-12) and written consent was collected before the surgery from the patients.

### Transcriptome sequencing

Total RNA was extracted using the Trizol reagent (Thermo Fisher, Waltham, MA, USA) following the manufacturer’s protocol. RNA integrity was evaluated using the Agilent 2100 Bioanalyzer (Agilent Technologies, Santa Clara, CA, USA). The total RNA that had a standard of RNA integrity number (RIN) ≥ 7.0, 28 S/18S ≥ 0.7 was subjected to RNA-Seq. The libraries were constructed using TruSeq Stranded mRNA LTSample Prep Kit (Illumina, San Diego, CA, USA) according to the manufacturer’s instructions. The libraries were sequenced on an Illumina HiSeq X Ten platform. QC data was shown in Additional file 1: Fig. S1.

### Illumina 850K (EPIC) DNA methylation array

Genomic DNA was extracted from synovial tissue with the MagMAX™-96 DNA Multi-Sample Kit (Thermo Fisher Scientific, Waltham, MA, USA) according to the manufacturer’s instructions. Genome-wide methylation was detected by the Illumina Human Methylation 850K Beadchip (Zhuoli Tech, Shanghai, China). DNA concentration and integrity were assessed by a NanoDrop 2000 spectrophotometer (Thermo Fisher Scientific, Waltham, MA, USA) and agarose gel electrophoresis, respectively. DNA was bisulfite-treated using the Zymo Research EZ DNA methylation-Glod Kits (Zymo Research, Irvine, CA, USA). Bisulfite-converted DNA was analyzed on an Illumina Infinium Methylation EPIC 850K BeadChip (Illumina). Microarray data were extracted, and the DNA methylation level was calculated using GenomeStudio Methylation Module v1.8 software (Version 2011.1) with default parameters. QC data was shown in Additional file 1: Fig. S1.

### The gene set enrichment analysis

Gene set enrichment analysis (GSEA) is used to determine the statistically significant differences between two groups about a defined set of genes. The analysis was conducted using the clusterProfiler package of R software, and the data set was from the Molecular Signatures Database v7.2 (MSigDB) downloaded from the GSEA-MSigDB website. The MSigDB is a database of gene sets for performing gene set enrichment analysis [9]. |NES|>1, *P* < 0.05, *FDR ≤ 0.25* were selected as the cut-off criteria indicating statistically significant differences. KEGG gene sets, C5: ontology gene sets are the target data sets for our study. The R codes were provided in Additional file 2.

### The weighted gene co-expression network analysis

Weighted gene co-expression network analysis (WGCNA) is used to construct the gene coexpression network and identify the functional modules [10]. The WGCNA was down by the WGCNA R software package [11]. Firstly, we normalized the samples first and then removed the outlier samples. The soft threshold power must be selected according to the standard scale-free networks, and all differential genes were calculated by a power function. Subsequently, the adjacency matrix was transformed into a topological overlap matrix (TOM), and the corresponding dissimilarity (1-TOM) was calculated. The dynamic tree cut method was performed to identify the module by hierarchically clustering genes [12]. The minModuleSize = 25. The Pearson correlation analysis was conducted to examine the relationship among gene modules to identify the module with the strongest association with RA.

### Identification of DEGs, DMPs, and DMGs

DEGs were performed using the DESeq R package [13]. Adjust *P*-value (False Discovery Rate) < 0.05 and |log2FoldChange| ≥1.5 were set as the threshold for significant differential expression. The ChAMP R package [14] was used to analyze the Methylation data. The criteria for the selection of DMPs are *FDR* < 0.05 and |Δβ| ≥ 0.1.

### Functional enrichment analysis

The MeDEGs were annotated by the GO functional enrichment analysis which included biological process (BP), molecular function (MF), cellular component (CC), and KEGG pathway analysis [15]. KEGG is a database resource for elucidating the genes’ function at the molecular and higher levels, including biochemical pathways [16]. The annotation and visualization were performed by the clusterProfiler R package [17]. The enrichment analysis was performed by a hypergeometric test. *P* < 0.05 was chosen as the cut-off criterion indicating a statistically significant difference. The R codes were provided in Additional file 2.

### The protein–protein interaction network construct and hub gene identified

The protein–protein interaction (PPI) network of MeDEGs was constructed by the STRING database. STRING is capable of fully describing user gene lists and functional genomic datasets and allows the creation and sharing of highly customized and enhanced protein-protein association networks [18]. The “minimum required interaction score” choose high confidence (0.7). The PPI network was displayed by Reactome functional interactions (FI) Cytoscape Plugin. Reactome FI Cytoscape Plugin can be used to find network patterns by the Reactome database [19].

### Correlation analysis between methylation level and mRNA expression

R software was used to explore the association between methylation level and expression by spearman correlation analysis. R ≥ 0.4, *P* < 0.05 was used as the criterion for screening correlation. The R codes were provided in Additional file 2.

## Results

### The results of the transcriptome and DNA methylation array

A total of 17,736 genes were detected in our transcriptome sequencing data. In our Illumina 850K DNA methylation array sequencing data, a total of 25,578 methylated genes and 755,852 methylation probes were detected.

### The GSEA of methylation-regulated expressed genes

**Fig. 2:**
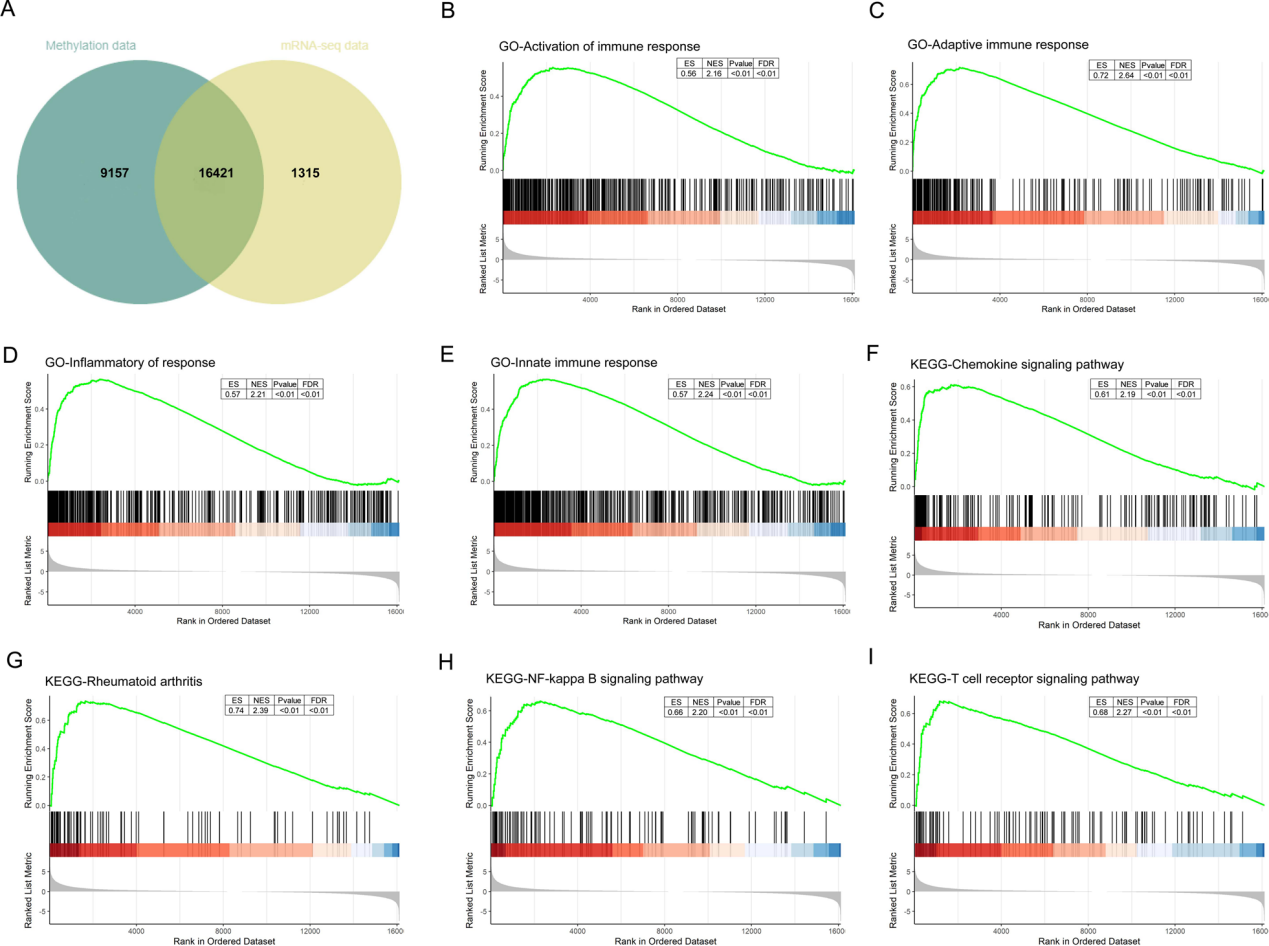
GSEA of methylation-regulated expressed genes. **A** Venn diagram of methylation-regulated expressed genes; **B**–**E** C5 (ontology gene sets); **C**–**I** KEGG sets; GSEA: Gene Set Enrichment Analysis. ES: enrichment score; NES: normalized enrichment score. The green line means enrichment profile. The red part of the heat map represents the genes are highly expressed in RA, blue part represents the genes are highly expressed in OA, the gray area of map represents the signal-to-noise ratio of each gene

The intersection of the two sets of sequencing data was used to obtain methylation-regulated expressed genes. A total of 16,421 methylation-regulated expressed genes were obtained. The Venn diagram is shown in Fig. 2A. The first part is the Enrichment score line graph: the score at the highest peak is the ES value of the gene set. In the second part, the black lines represent gene positions in the sorted gene table. The leading edge subset is the part of the genes corresponding to the origin to the peak ES of the green curve, indicating the genes that have a major contribution to the enrichment. The third part is the distribution of the rank values of all genes after sorting. The genes corresponding to the red part of the heat map are highly expressed in RA, the genes corresponding to the blue part are highly expressed in OA, and the signal-to-noise ratio corresponding to each gene is shown in the gray area map [20]. The GSEA shown that these genes are associated with activation of immune response, adaptive immune response, Inflammatory response, Innate immune response in C5 (ontology gene sets). For KEGG analysis, these genes are associated with chemokine signaling pathway, rheumatoid arthritis, NF-kappa B signaling pathway, T cell receptor signaling pathway. Normalized Enrichment Score (NES) and FDR were presented in the upper right corner of the plot (Fig. 2B–I).

### The WGCNA of methylation-regulated expressed genes

**Fig. 3:**
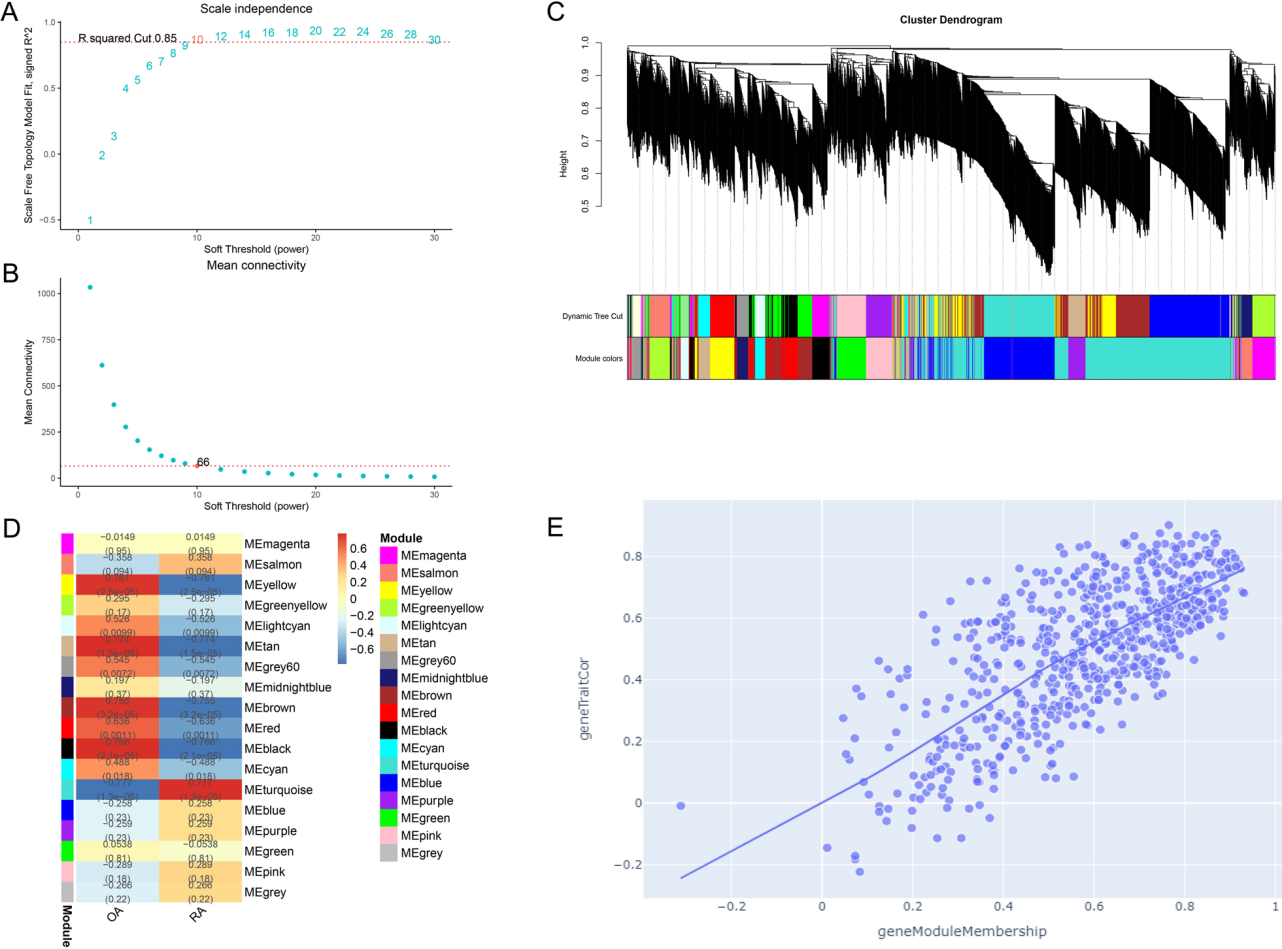
Identification of RA-related module. **A** Graph of scale independence, **B** graph of mean connectivity, **C** cluster dendrogram of the co-expression network modules, **D** cluster plot analysis of the relationship between RA and modules. **E** Scatter plot analysis of the turquoise module

The soft-thresholding power was ten which was determined based on R squared cut of 0.85 (Fig. 3A, B). Eighteen modules were identified based on average hierarchical clustering and dynamic tree clipping (Fig. 3C). The turquoise module exhibited the strongest correlation with RA (R = 0.78, *P* = 1.27 × 10^− 05^) (Fig. 3D). 660 genes were screened in the turquoise module for subsequent analysis (Fig. 3E).

### Identification of DEGs, DMGs, and DMPs

**Fig. 4:**
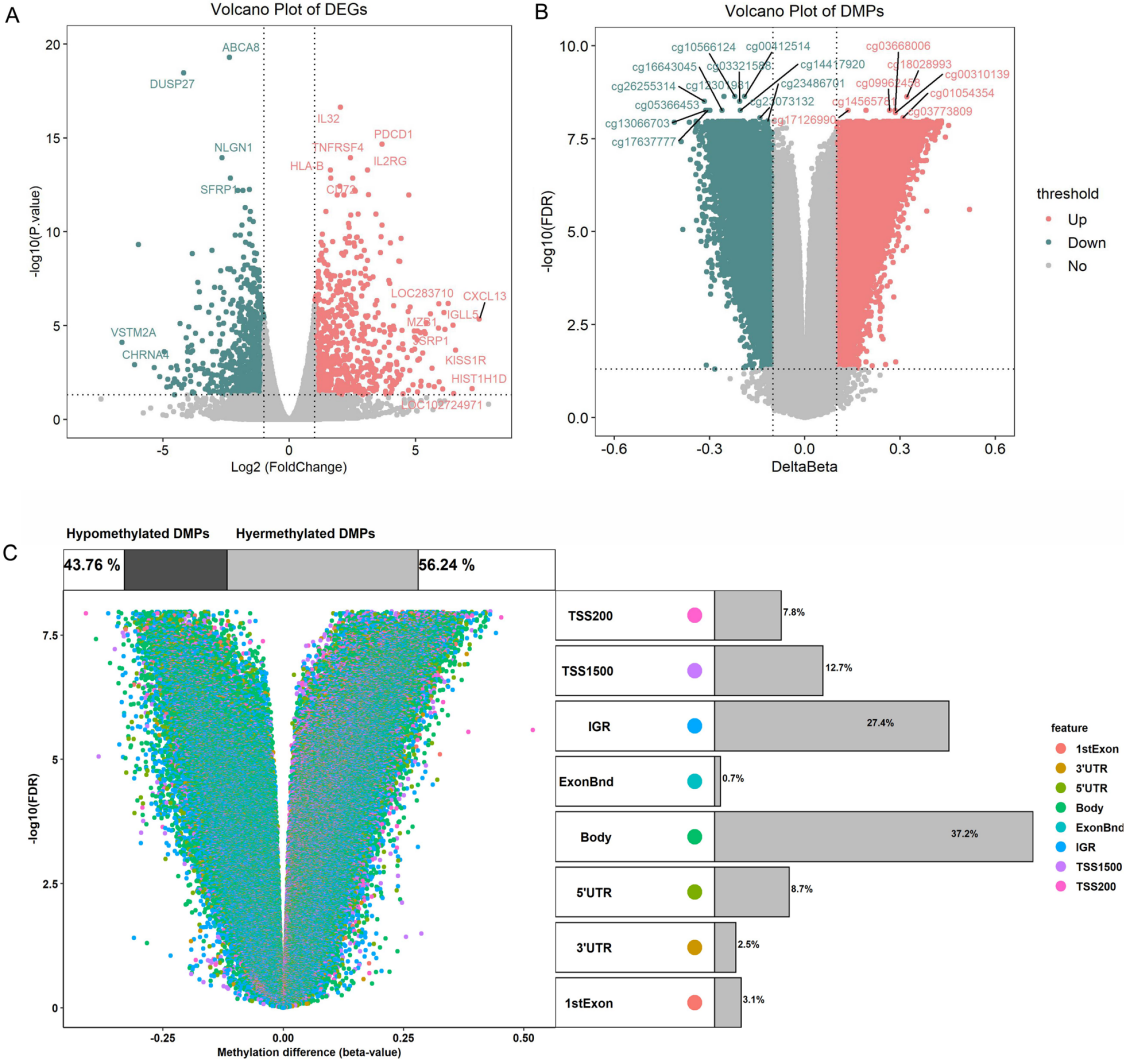
Differentially expressed genes and differentially methylated probes in RA. **A** The volcano plot of DEGs; **B** the volcano plot of DMPs. The red dots represent up-regulation and the green dots represent down-regulation; **C** the Distribution of DMPs

In our transcriptome sequencing data, 851 DEGs and 16250 DMGs were identified. The volcano plots were shown in Fig. 4A and B. Differential methylation probes are distributed differently in each region, among which the promoter region (1stExon,5’UTR, TSS1500, TSS200) accounts for about 32.3% (Fig. 4C).

### The GO and KEGG analysis of MeDEGs

**Fig. 5:**
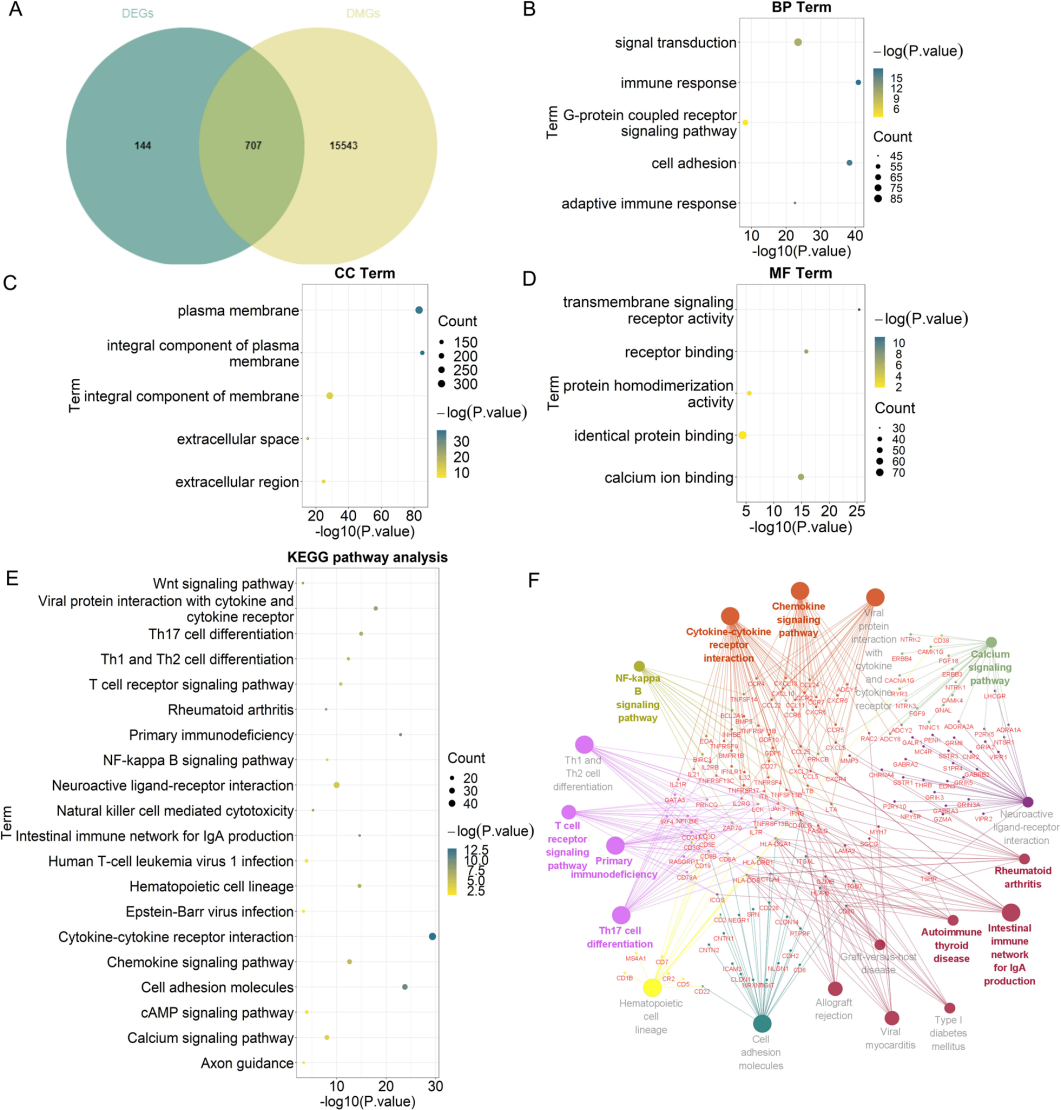
The GO and KEGG pathway analysis of MeDEGs. **A** The Venn diagram of MeDEGs; **B** BP terms; **C** CC terms; **D** MF terms; **E**, **F** KEGG pathway enrichment terms. BP: biological process; MF: molecular function; CC: cell component; MeDEGs: methylation-regulated differentially expressed genes; KEGG: Kyoto Encyclopedia of Genes and Genomes; GO: Gene Ontology. The size of the dots represent the number of enriched genes, and the color represents the value of *P* value. The bigger the dot, the higher the column, the more genes are enriched. The darker the color, the smaller the *P* value

The intersection of DEGs and DMGs was used to obtain MeDEGs. A total of 707 MeDEGs were obtained. The Venn diagram is shown in Fig. 5A. The results of GO functional enrichment analysis showed that MeDEGs were enriched in signal transduction, cell adhesion, G-protein coupled receptor signaling pathway for BP (Fig. 5B), enriched in plasma membrane, integral component of membrane, integral component of plasma membrane for CC (Fig. 5C), and enriched in identical protein binding, calcium ion binding, protein homodimerization activity for MF (Fig. 5D). The KEGG pathway analysis showed that the MeDEGs were enriched in cytokine–cytokine receptor interaction, calcium signaling pathway, T cell receptor signaling pathway, NF-kappa B signaling pathway, Rheumatoid arthritis (Fig. 5E, F).

### The PPI network analysis and hub genes screened

**Fig. 6:**
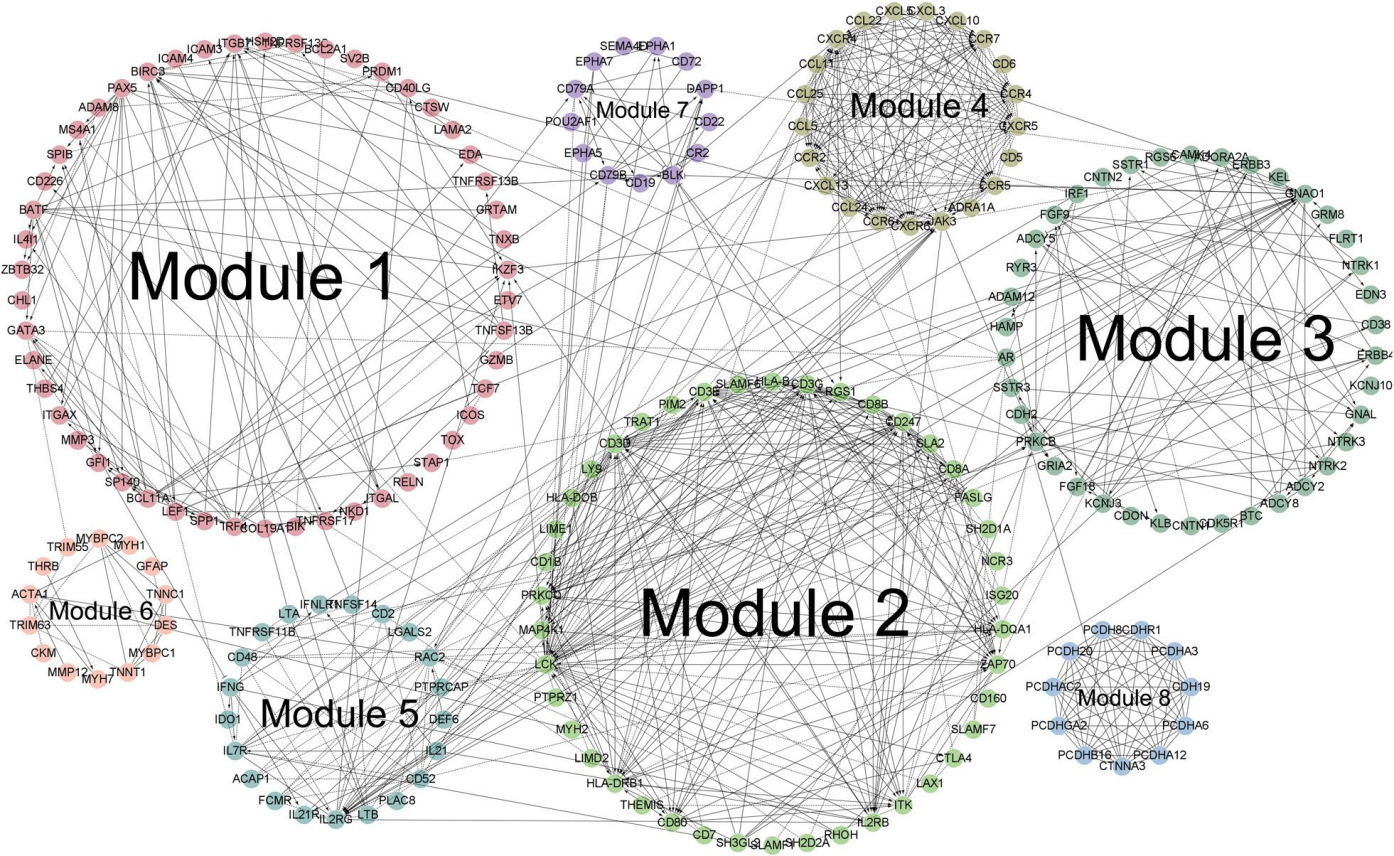
Protein–protein interaction network. Digital from ‘1’ to ‘8’ represent different modules constructed by Reactome functional interactions Cytoscape Plugin. Each edge between any two proteins (dots) indicates an interaction

Based on the STRING database, the PPI network contains 706 nodes and 882 edges, and the enrichment *P-value* < 1.0 × 10^− 16^. With the Reactome FI Cytoscape Plugin, based on the range of more than 10 genes, a total of 8 modules were screened out (Fig. 6). Genes in module 1 are mainly related to pathways including Intestinal immune network for IgA production, NF-kappa B signaling pathway. Genes in module 2 are mainly related to pathways including Th17 cell differentiation, T cell receptor signaling pathway, Th1 and Th2 cell differentiation. Genes in module 5 are mainly related to pathways including JAK-STAT signaling pathway, Inflammatory bowel disease. Genes in module 6 and module 8 are mainly related to pathways including Wnt signaling pathway.

Based on the turquoise module analyzed by WGCNA and the network modules constructed by PPI, we identified SPP1, RGS1, RAC2, MMP3, IL32, CD52, CCL5 as our hub genes. Detailed information on genes and probes can be found in Table 1.

**Table 1:** Detailed information on genes and probes

ProbeID	Gene	FDR	deltaBeta	Methylation_status	Group
cg02549628	SPP1	1.91 × 10^− 06^	− 0.17	Hypomethylated	TSS1500
cg20261167	SPP1	1.90 × 10^− 03^	− 0.12	Hypomethylated	1stExon
cg15460348	SPP1	2.57 × 10^− 03^	− 0.11	Hypomethylated	1stExon
cg10718027	RGS1	2.25 × 10^− 08^	− 0.28	Hypomethylated	TSS200
cg02586212	RGS1	6.91 × 10^− 08^	− 0.23	Hypomethylated	1stExon
cg10861751	RGS1	9.57 × 10^− 08^	− 0.21	Hypomethylated	TSS200
cg08235798	RAC2	2.91 × 10^− 04^	− 0.11	Hypomethylated	TSS1500
cg16466334	MMP3	9.53 × 10^− 03^	0.12	Hypermethylated	TSS200
cg01594685	IL32	7.42 × 10^− 05^	0.10	Hypermethylated	TSS1500
cg23403079	CD52	3.91 × 10^− 07^	− 0.22	Hypomethylated	TSS200
cg08572767	CD52	1.00 × 10^− 06^	− 0.19	Hypomethylated	TSS200
cg07188645	CCL5	4.22 × 10^− 04^	− 0.12	Hypomethylated	TSS1500
cg19411729	CCL5	5.14 × 10^− 03^	− 0.10	Hypomethylated	TSS1500

### Correlation analysis between methylation level and mRNA expression

**Fig. 7:**
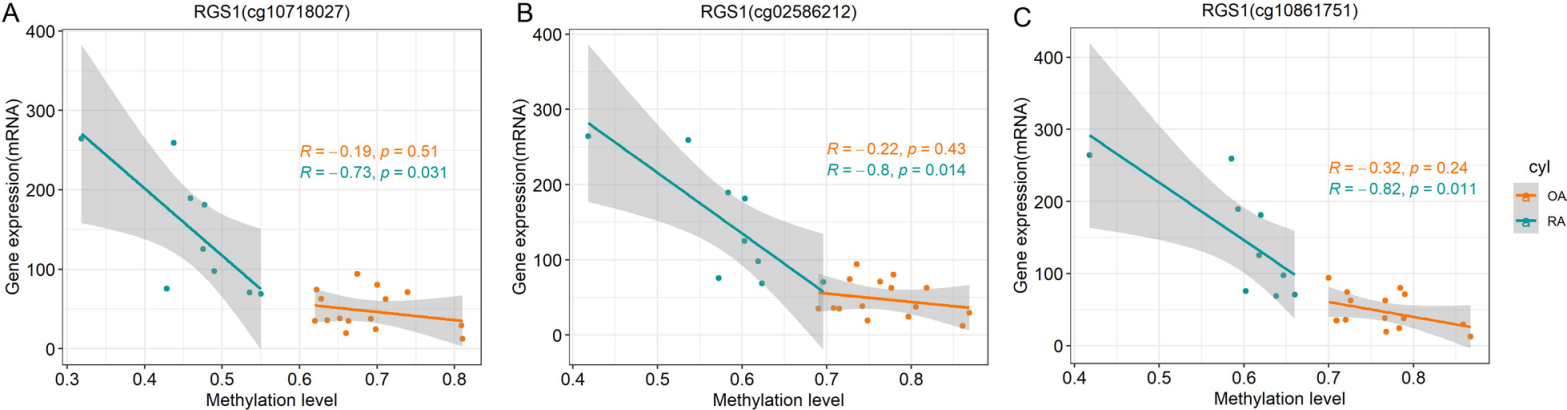
DNA methylation/mRNA correlation plots for differential methylation positions/genes. *R*: spearman correlation coefficient

Spearman correlation analyses showed significant associations between DNA methylation levels of 3 probes and mRNA expressions of RGS1. RGS1 (cg10718027) (R = − 0.73), RGS1(cg02586212) (R = − 0.80), RGS1(cg10861751) (R = − 0.82) (Fig. 7A–C). Other correlations are shown Additional file 3: Fig. S3.

## Discussion

RA is an autoimmune disease, its occurrence is a complex biological process, including the abnormal expression of immune-related genes, the activation of immune-related pathways [21]. Thus, it is important to identify the genes and signaling pathways involved in the pathogenic process in RA. Sequencing or microarray combined with bioinformatics analysis has become an effective method to reveal the molecular mechanism of numerous diseases. Huo et al. [22] determined that abnormal methylation of CDC20 and CCNA2 may be effective in predicting the prognosis of RA with microarray combined with bioinformatics analysis. For RA, some studies combing microarray with bioinformatics analysis have revealed differential methylation signals at S100A6 and EFCAB4B promoter regions in the whole blood [23], CD86, RAB20, XAF1, FOLR3, LTBR, KCNH8, DOK7, PDGFA, PITPNM2, and CELSR1 in B cells [24], and IFN related genes (IFIT1, IRF7, MX1, OAS1, USP18, RSAD2, IFI44L) in CD4+ T cells [25]. However, until now, the methylation of RA-related genes are still insufficient. Identification of methylation-regulated differentially expressed genes (MeDEGs) of RA based on high-throughput data will be of profound significance for clarifying the role of methylation and identifying candidate directions for future research.

In this present research, we identified 17,736 genes in our transcriptome sequencing data, a total of 25,578 methylated genes, and 755,852 methylation probes in our Illumina 850K BeadChip data. After the intersection of the two sets of data, 16,421 methylation-regulated expressed genes were found, and then, GSEA was used to perform these genes. The results of GSEA showed that these genes are associated with activation of immune response, adaptive immune response, inflammatory response, innate immune response in C5 (ontology gene sets). For KEGG analysis, these genes are associated with chemokine signaling pathway, rheumatoid arthritis, NF-kappa B signaling pathway, T cell receptor signaling pathway.

With WGCNA, 660 genes in turquoise module were strongly associated with RA. Furthermore, we screened 851 DEGs and 16,250 DMGs. We took the intersection of the DMGs and DEGs to obtain 188 MeDEGs. The GO functional enrichment analysis showed that MeDEGs were enriched in signal transduction, cell adhesion, G-protein coupled receptor signaling pathway for BP, enriched in plasma membrane, integral component of membrane, integral component of plasma membrane for CC, and enriched in identical protein binding, calcium ion binding, protein homodimerization activity for MF. The KEGG pathway analysis showed that the MeDEGs were enriched in cytokine-cytokine receptor interaction, calcium signaling pathway, T cell receptor signaling pathway, NF-kappa B signaling pathway, Rheumatoid arthritis. The calcium signaling pathway plays an important role in autoimmune diseases such as RA [26, 27]. Mutations of the genes encoding T cell receptor signaling molecules, such as ZAP-70, can cause T-cell mediated autoimmune diseases, including RA [28]. GO and KEGG bioinformatics analysis showed that the function and involved pathways of MeDEGs are closely related to RA.

Based on the turquoise module analyzed by WGCNA and the network modules constructed by PPI, we screened out SPP1, RGS1, RAC2, MMP3, IL32, CD52, CCL5 for further study. SPP1 (cg02549628), (cg20261167), (cg15460348); RGS1 (cg10718027), (cg02586212), (cg10861751); RAC2 (cg08235798); MMP3(cg16466334); IL32 (cg01594685); CD52 (cg23403079), (cg08572767); CCL5 (cg07188645), (cg19411729). Methylation of these gene loci, which occurs near the promoter, is more likely to affect gene expression. Spearman correlation analysis was used to analyze the relationship between gene methylation levels and mRNA expression. The genes and probes with the highest correlation were RGS1(cg10718027), RGS1(cg02586212), RGS1(cg10861751). Regulator of G protein signaling 1 (RGS1) is has been reported to be associated with multiple cancers [29]. MicroRNA-376b-3p can target RGS1 mRNA to inhibit the development of osteosarcoma [30], and RGS1 expression desensitizes G-protein-coupled receptor signaling and is associated with poor prognosis in multiple myeloma [31]. Prolonged inflammation of the synovial membrane in RA can lead to the formation of pannus. The persistent aggressiveness of the pannus makes RA a tumor-like disease [32]. RGS1 has been extensively studied in tumors, making its study in RA very promising. Most importantly, inhibition of RGS1 expression can inhibit inflammatory response and angiogenesis in CIA rats by inhibiting TLR signaling pathway, thus providing a new therapeutic target for RA treatment. Our study further confirms the important role of the RGS1 gene in RA.

In conclusion, we reported possible methylation-regulated differentially expressed biomarkers and revealed the molecular functions of screened genes involved in RA by integrated bioinformatics analysis. These results guide to further studying the underlying mechanisms of RA development. We identified RGS1 that may serve as novel biomarkers and potential targets for accurate diagnosis and treatment strategies for RA. Further experiments are warrant to verify these biomarkers and to explore the specific mechanisms underlying RA.

## Supplementary Information

**Additional file 1:** Data Quality Control (QC).

**Additional file 2:** R code.

**Additional file 3:** Correlation between mRNA expression and methylation level of other genes.
