# Supplementary figures and images for: Identification of DNA methylation-regulated differentially expressed genes in RA by integrated analysis of DNA methylation and RNA-Seq data
Source: J Transl Med. 2022 Oct 22;20:481. doi: 10.1186/s12967-022-03664-5 (PMC9588210; doi:10.1186/s12967-022-03664-5)

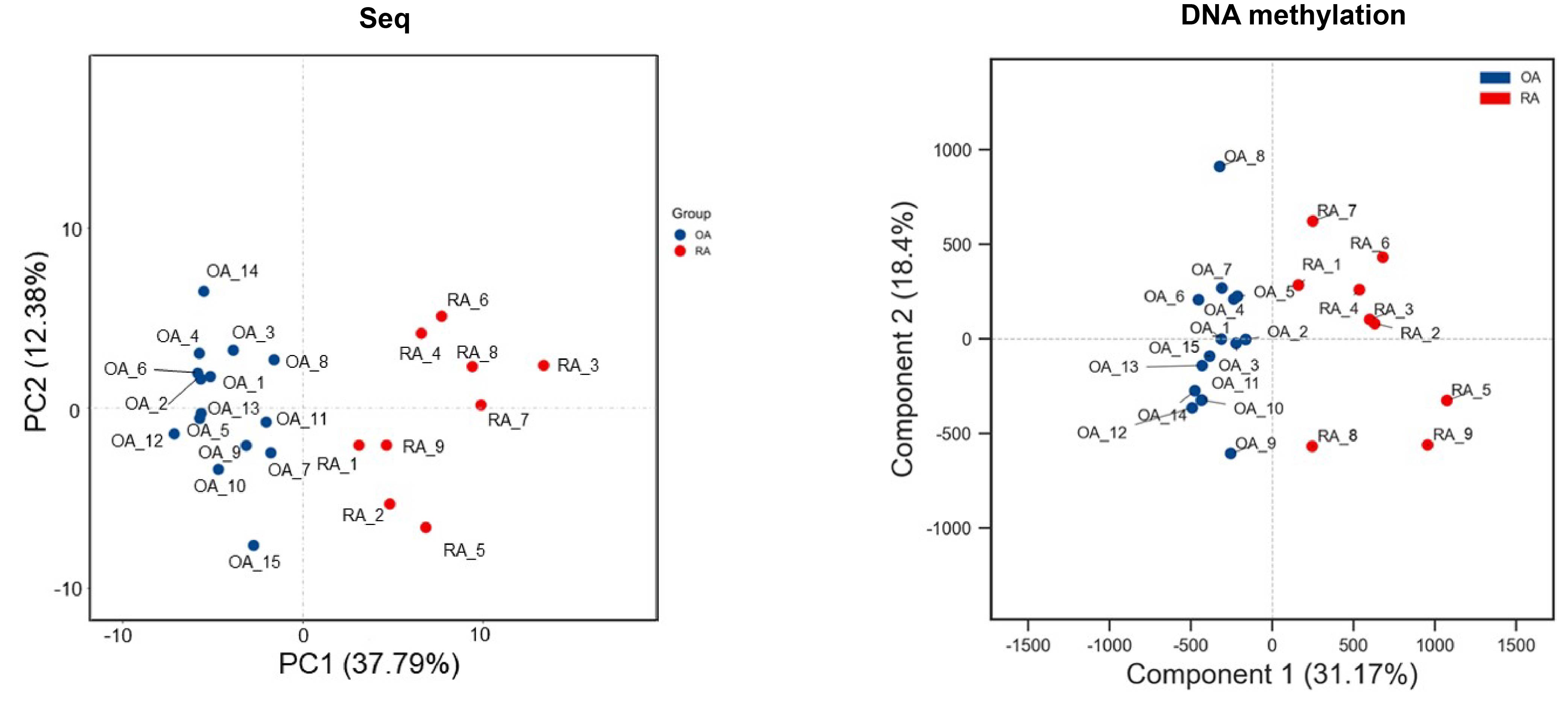

Supplement: Supplementary file 1 — Additional file 1: Data Quality Control (QC). [file 12967_2022_3664_MOESM1_ESM.jpg]

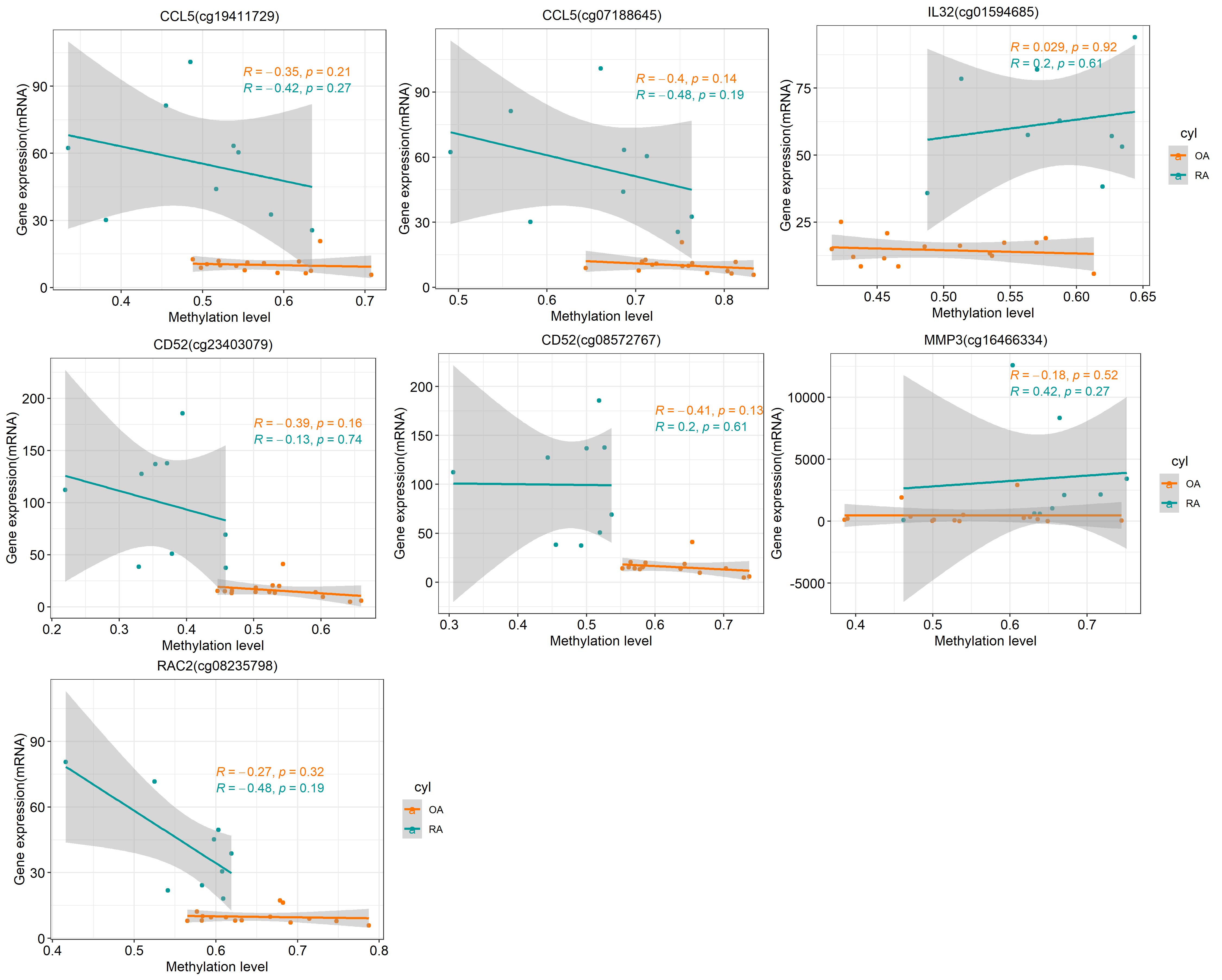

Supplement: Supplementary file 3 — Additional file 3: Correlation between mRNA expression and methylation level of other genes. [file 12967_2022_3664_MOESM3_ESM.jpg]
